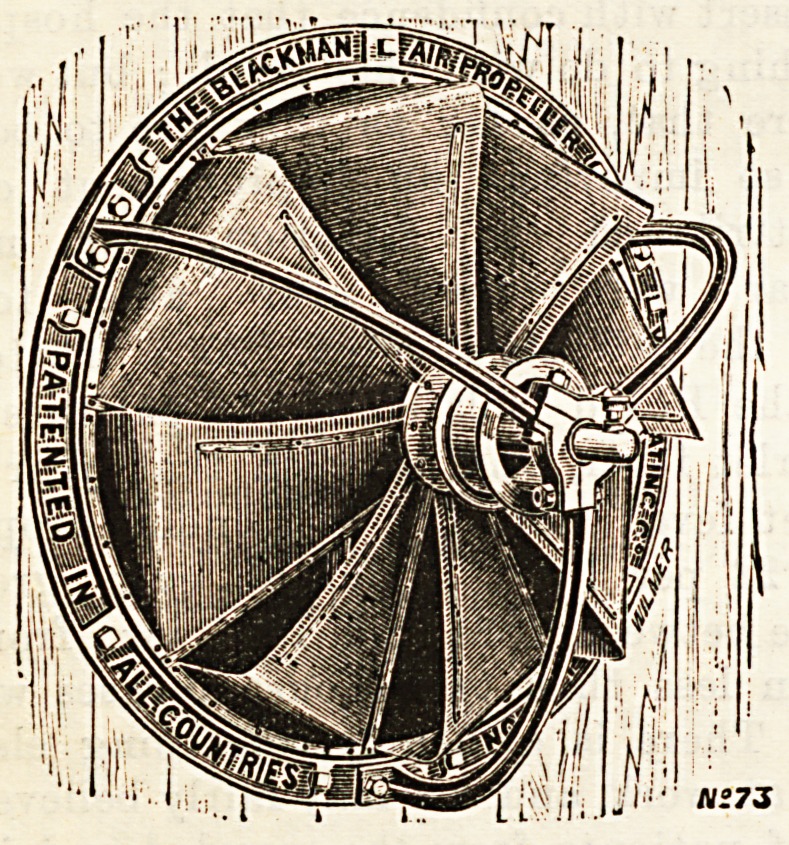# The Blackman Rapid Drying System

**Published:** 1895-05-25

**Authors:** 


### PRACTICAL DEPARTMENTS.

### THE BLACKMAN RAPID DRYING SYSTEM.

The best and most satisfactory method of drying clothes is naturally in the open air, by the action of sun and wind; but in towns and in large laundries, both on account of the amount of work to be done and of space limit, as well as in this country the uncertainty of weather, and in London and other large cities the all-pervading dirt of the atmosphere, artificial means have to be resorted to, and apparatus of more or less elaborate design will nowadays be found in every laundry. The plan most largely adopted at present is that of heated closets, fitted with draw-out horses, upon which are placed the clothes in process of drying. There are many varieties of this arrangement, and it is usually found to answer very satisfactorily; but a better way is undoubtedly to effect the process by means of streams of hot air propelled by fans through the drying chamber.

At the Laundry Exhibition held last autumn at the Agricultural Hall this system was shown in work; the Blackman Ventilating Company having a drying-room erected there for inspection. The procedure under this plan is as follows: The drying-room is divided through the centre by a partition, "at one end a Blackman warmer raises to about 140 degrees Fahr. the large volume of air continually blown through it by a Blackman fan, and the hot air is directed by a swing-door into either or both halves of the room at pleasure; the linen is hung parallel with the partition on rods fixed to the ceiling, and the floor is entirely clear throughout. The same fan at the same time blows cool air through either of two roomy passages, as well as alongside the heater into either half of the drying-room at pleasure. A swing-door is fitted in each of these cool air passages, as well as in front of the in-let opening through which the warm air enters from the air warmer." The swing-doors are governed by a cord outside the chamber, enabling the temperature to be easily regulated; a high temperature used for the drying, and the room then cooled down to allow of the attendant removing and replacing the linen. By this division of the closet the drying process is made continuous; one-half being cleared and refilled at a time.

This method of drying is equally well suited to every kind of material, the results so far as fabric is concerned being extremely good. It may readily be seen that the continuous passing of currents of air over stuffs to be dried is a much nearer approach to the natural process than stewing at a high temperature in a closet where very little change of atmosphere is possible. An important question is naturally the practical one of comparative cost; in many instances, the most efficient way of carrying out any particular work is almost unattainable on this account, the increased expense prohibitory to its general utilisation. In the present case, inquiries made show that in practice no such drawback exists, and as from the sanitary and hygienic point of view the "Rapid Drying" process is one to be recommended, so also on economic grounds its adoption may be advocated. For hospital laundries it is almost certain to become widely accepted as an improvement upon other and less airy methods.

The illustration shows the shape of fan or propeller known as the "Blackman." These fans are also largely in use in mills, factories, &c., for ventilation purposes, where their application for the clearing of the air and the removal of dust and steam is attended with much success and benefit to the workers and the machinery.

## Figures and Tables

**Figure f1:**